# Synthesis and *E*/*Z *Configuration Determination of Novel Derivatives of 3-Aryl-2-(benzothiazol-2'-ylthio) Acrylonitrile, 3-(Benzothiazol-2'-ylthio)-4-(furan-2''-yl)-3-buten-2-one and 2-(1-(Furan-2''-yl)-3'-oxobut-1''-en-2-ylthio)-3-phenylquinazolin-4(3*H*)-one

**DOI:** 10.3390/molecules16076129

**Published:** 2011-07-20

**Authors:** Fatima Al-Omran, Rafat M. Mohareb, Adel Abou El-Khair

**Affiliations:** 1 Department of Chemistry, Faculty of Science, Kuwait University, P.O. box 12613, safat 13060, Kuwait; 2 Department of Chemistry, Faculty of Science, Cairo University, Giza, A.R., Egypt; 3 Department of Organic Chemistry, Faculty of Pharmacy, October University for Modern Sciences and Arts, Elwahaat Road, October City, A.R., Egypt

**Keywords:** benzothiazole, 2-mercaptobenazothiazole, 3-phenylquinazolin-4(3*H*)-one, methyl vinyl ketone, acrylonitrile

## Abstract

Knoevenagel condensation of 2-(benzothiazol-2-ylthio) acetonitrile (**2**) with either furan-2-carbaldehyde or thiophene-2-carbaldehydes leads to *E-i*somers **4a–b** exclusively, while the condensation of the compound **2** with benzaldehyde or *para*-substituted benzaldehydes with an electron-donating group afforded *E/Z* mixtures **4c–e** with preferentially formation of the *E*-isomer. Condensation of furan-2-carbaldehyde (**3a**) with either 1-(benzothiazol-2'-ylthio) propan-2-one (**5**) or 2-(2'-oxo propylthio)-3-phenyl-quinazolin-4(3*H*)-one (**9**) leads exclusively to the *Z-*isomers of **6** and **10**, respectively. The structures of the newly synthesized compounds were elucidated by elemental analyses, ^1^H-NMR and ^13^C-NMR spectra, COSY, HSQC, HMBC, NOE, MS and X-ray crystallographic investigations.

## 1. Introduction

The benzothiazole nucleus is a highly important scaffold for drug development, which has been reported to show good biological activities ranging from anti-microbial [1], anti-inflammatory [1], antibacterial [2], antitumor [3] and anticancer [4,5] to antifungal [6]. On other hand, quinazolin-4-(3*H*)-ones substituted in the 3-position with a heterocyclic system are attracting the attention of chemists, because of their biological activities ranging from antibacterial, antimicrobial, antifungal, anticonvulsive, sedative, anti-inflammatory, to hypnotic and CNS depression [7,8,9,10,11,12]. Furthermore, several other publications have pointed out the value of 3-arylacylonitriles with either triazole [13] or benzimidazole [14] substituents in position 2 of the acrylonitrile also have good cytotoxic activity on human cancer cells and are antibacterial agents [14]. It was recently reported that 2-acetyl-3-(6-methoxybenzothiazo)-2-yl-amino-acylonitrile (AMBAN) possesses significant anti-proliferative activity and is a potent inducer programmed cell death in human leukemia cells [5].

Based on the abovementioned effects of benzothiazoles, 3-arylacrylonitriles and quinazolin-4-(3*H*)-ones a series of novel 3-arylacrylonitriles with 2-mercaptobenzothiazoles moiety in the position 2- of the acrylonitrile were synthesized to obtain new potential biologically active agents. On other hand, a series of 4-aryl-3-buten-2-ones with either 2-mercaptobenzothiazole or quinazolin-4-(3*H*)-one rings in the 3-position of 4-aryl-3-buten-2-one were synthesized. The structures of the newly synthesized compounds have been established by x-ray diffraction studies and on the basis of their spectral data.

## 2. Result and Discussion

**Scheme 1:**
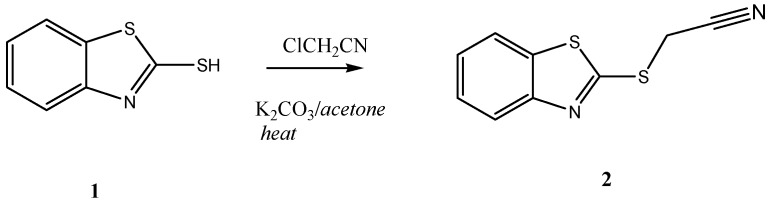
Synthesis of **2**.

**Figure 1:**
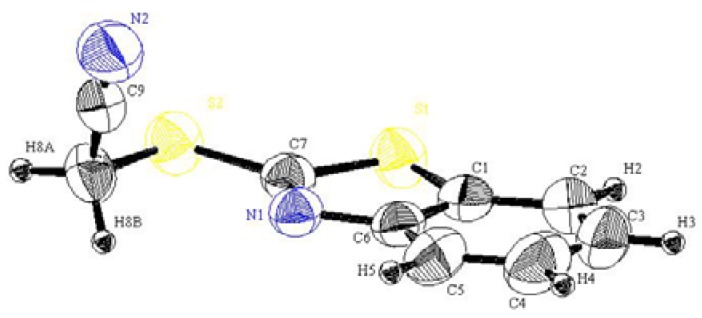
Perspective view and atom labeling of the X-ray structure of compound **2**.

In continuation to our recent research programme dealing with the synthesis of heterocyclic systems, particularly those containing the 2-mercaptobenzothiazole moiety [15,16], 1-(benzothiazol-2-yl-thio)acetonitrile (**2**) was readily prepared in an excellent yield by treatment of 2-mercapto-benzothiazole (**1**) with α-chloroacetonitrile in refluxing acetone containing anhydrous potassium carbonate (*cf*. Scheme 1). The structure of **2** was unambiguously confirmed by X-ray crystallography [17] as well as on the basis of its spectral data (*cf*. Figure 1 and Table 1, Table 2, Table 3).

**Table 1:** Bond lengths (Å) for compound **2**.

atom	atom	distance	atom	atom	distance
S1	C1	1.730(3)	S1	C7	1.741(3)
S2	C7	1.747(3)	S2	C8	1.802(3)
S3	C10	1.732(3)	S3	C16	1.750(3)
S4	C16	1.745(3)	S4	C17	1.806(3)
S5	C19	1.731(3)	S5	C25	1.748(3)
S6	C25	1.747(3)	S6	C26	1.807(3)
N1	C6	1.400(4)	N1	C7	1.293(3)
N2	C9	1.136(5)	N3	C15	1.396(4)
N3	C16	1.292(3)	N4	C18	1.131(6)
N5	C24	1.402(3)	N5	C25	1.296(4)
N6	C27	1.136(6)	C1	C2	1.391(5)
C1	C6	1.404(4)	C2	C3	1.375(5)
C3	C4	1.388(5)	C4	C5	1.370(5)
C5	C6	1.384(4)	C8	C9	1.460(5)
C10	C11	1.392(4)	C10	C15	1.403(3)
C11	C12	1.366(4)	C12	C13	1.389(4)
C13	C14	1.373(5)	C14	C15	1.386(4)
C17	C18	1.447(5)	C19	C20	1.394(4)
C19	C24	1.400(4)	C20	C21	1.373(5)
C21	C22	1.391(4)	C22	C23	1.383(4)
C23	C24	1.386(4)	C26	C27	1.446(5)

**Table 2:** Bond angles (o) for compound **2**.

atom	atom	atom	angle	atom	atom	atom	angle
C1	S1	C7	88.34(12)	C7	S2	C8	98.36(13)
C10	S3	C16	88.53(12)	C16	S4	C17	98.83(13)
C19	S5	C25	88.36(12)	C25	S6	C26	99.08(12)
C6	N1	C7	109.3(2)	C15	N3	C16	109.99(19)
C24	N5	C25	109.60(18)	S1	C1	C2	129.17(19)
S1	C1	C6	109.9(2)	C2	C1	C6	121.0(3)
C1	C2	C3	117.7(3)	C2	C3	C4	121.5(4)
C3	C4	C5	120.9(3)	C4	C5	C6	118.9(3)
N1	C6	C1	114.9(2)	N1	C6	C5	125.1(3)
C1	C6	C5	120.0(3)	S1	C7	S2	117.68(14)
S1	C7	N1	117.6(2)	S2	C7	N1	124.7(2)
S2	C8	C9	112.7(3)	N2	C9	C8	179.6(3)
S3	C10	C11	129.56(18)	S3	C10	C15	109.71(19)
C11	C10	C15	120.7(3)	C10	C11	C12	118.5(3)
C11	C12	C13	121.1(3)	C12	C13	C14	121.0(3)
C13	C14	C15	119.0(3)	N3	C15	C10	115.0(3)
N3	C15	C14	125.2(2)	C10	C15	C14	119.8(3)
S3	C16	S4	118.30(13)	S3	C16	N3	116.8(2)
S4	C16	N3	124.92(18)	S4	C17	C18	112.2(3)
N4	C18	C17	178.8(4)	S5	C19	C20	128.9(2)
S5	C19	C24	110.11(16)	C20	C19	C24	121.0(3)
C19	C20	C21	117.9(3)	C20	C21	C22	121.5(3)
C21	C22	C23	120.9(3)	C22	C23	C24	118.4(3)
N5	C24	C19	114.8(2)	N5	C24	C23	124.9(2)
C19	C24	C23	120.3(2)	S5	C25	S6	117.87(15)
S5	C25	N5	117.12(16)	S6	C25	N5	125.00(16)
S6	C26	C27	112.65(17)	N6	C27	C26	178.8(3)

**Table 3:** Bond lengths involving hydrogen’s (Å) for compound **2**.

atom	atom	distance	atom	atom	distance
C2	H2	0.930	C3	H3	0.930
C4	H4	0.930	C5	H5	0.930
C8	H8A	0.970	C8	H8B	0.970
C11	H11	0.930	C12	H12	0.930
C13	H13	0.930	C14	H14	0.930
C17	H17A	0.970	C17	H17B	0.970
C20	H20	0.930	C21	H21	0.930
C22	H22	0.930	C23	H23	0.930
C26	H26A	0.970	C26	H26B	0.970

**Scheme 2:**
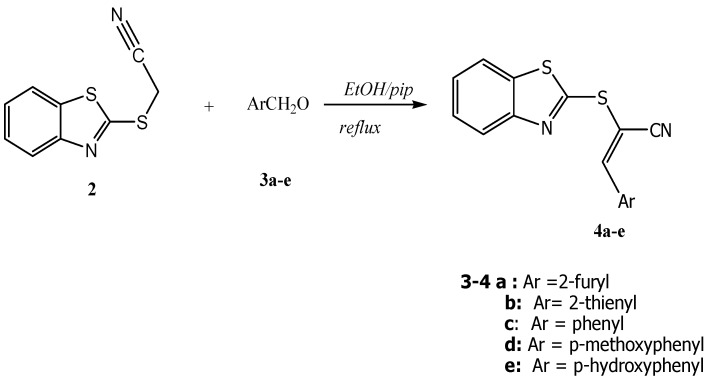
Syntheses of **4**.

Knoevenagel condensation of the ethanolic solution of 1-(benzothiazol-2-yl-thio) acetonitrile (**2**) with either hetero-2-carbaldehydes **3a–b** or benzaldehyde or *para*-substituted benzaldehydes **3c–e** with an electron-donating group at the *para*-position, as depicted in Scheme 2. The reactions were carried out in the presence of a catalytic amount of piperidine at the reflux temperature leading to novel 3-aryl-2-(benzothiazol-2'-ylthio)acrylonitriles **4a–e** (*cf*. Scheme 2).

**Figure 2:**
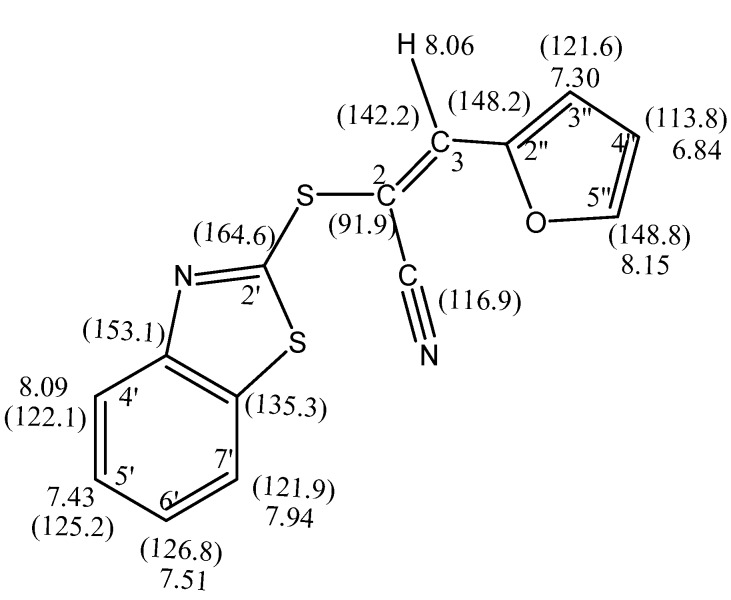
The complete assignment of H^1^ and ^13^C chemical shift for **4a** based on the COSY, HSQC and HMBC experiments.

The new 3-aryl-2*-*(benzothiazol-2'-ylthio)acrylonitrile derivatives **4a–e** which were formed are highly conjugated systems containing heteroaromatic or phenyl rings, or *para*-substituted phenyl rings with an electron-donating group in the *para*- position such as –OCH_3_ or –OH. One the other hand, all systems are linked to the benzothiazole nuclei through an *S* linkage at the α-vinylic carbon. The formation of a carbon-carbon double bond usually lead to the creation of acrylonitriles with either *E* or *Z *configuration or a mixture of *E *and *Z* isomers (*cf*. Scheme 2). The yields are based on isolated products and the *E*/*Z* ratio was affected by the kind of substitution of the aryl aldehydes **3a–e**. The *E*/*Z *ratio was determined on the basis of the ^1^H-NMR spectra of the products. The structures of isolated *S*-alkylated acrylonitrile products **4a–e** were confirmed on the basis of elemental analysis and spectral data. The mass spectrum of **4a** revealed a molecular ion peak (M^+^) with *m*/*z* 284. The chemical shifts of protons for **4a** were assigned using the COSY (correlation spectroscopy) measurement which provided the proton-proton couplings. The ^1^H-NMR revealed in addition to an aromatic multiplet, a singlet signal for a vinylic proton (H-3), that appears at δ_H_ 8.06 ppm. Moreover, the chemical shifts of carbons for compound **4a** were assigned using HSQC (Heteronuclear Single Quantum Coherence) and HMBC (Heteronuclear Multiple Bond Coherence) measurements. The ^13^C-NMR spectrum for **4a** is characterized by two signals at δ_C_ 142.2 and δ_C_ 91.9 ppm for the carbon-carbon double bond group. The ^13^C signal of the β carbon at the higher frequency coupled with a proton, while the ^13^C signal of the α-carbon appears at lower frequency (*cf.* Figure 2).

**Figure 3:**
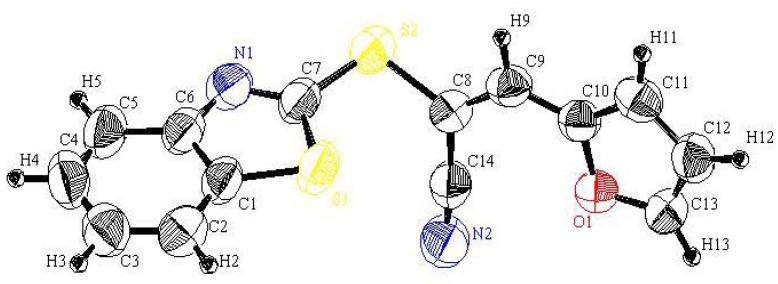
Perspective view and atom labeling of the X-ray structure of **4a**.

Nuclear Overhauser Effect (NOE) experiments were run to establish the stereo-orientation for acrylonitrile derivatives **4** as either *E* or *Z* isomers. NOE experiments for **4a** showed an enhanced triplet signal (H-4'') at δ_H_ 6.84 ppm upon irradiating the doublet signal (H-5'') at δ_H_ 8.15 ppm. On irradiating the doublet signal (H-3”) at δ_H_ 7.30 ppm both the triplet signal (H-4”) and the singlet signal (H-3) at δ_H_ 6.84 and 8.06 ppm, respectively, were enhanced, while the benzothiazole protons showed no effect, confirming that the benzothiazol-2-ylthio moiety and 2'-furyl groups are on opposite sides of the double bond as required by the *E-*form. The structure of **4a** was unambiguously confirmed by X-ray crystallography [18] (*cf*. Figure 3 and Table 4, Table 5, Table 6).

**Table 4:** Bond lengths (Å) for compound **4a**.

atom	atom	distance	atom	atom	distance
S1	C1	1.727(3)	S1	C7	1.747(3)
S2	C7	1.762(3)	S2	C8	1.770(3)
O1	C10	1.381(3)	O1	C13	1.352(4)
N1	C6	1.391(4)	N1	C7	1.298(4)
N2	C14	1.136(4)	C1	C2	1.406(5)
C1	C6	1.403(4)	C2	C3	1.363(5)
C3	C4	1.382(6)	C4	C5	1.355(7)
C5	C6	1.387(5)	C8	C9	1.349(4)
C8	C14	1.438(4)	C9	C10	1.401(4)
C10	C11	1.356(4)	C11	C12	1.403(4)
C12	C13	1.322(4)			

**Table 5:** Bond lengths involving hydrogen's (Å) for compound **4a**.

atom	atom	distance	atom	atom	distance
C2	H2	0.93	C3	H3	0.93
C4	H4	0.93	C5	H5	0.93
C9	H9	0.93	C11	H11	0.93
C12	H12	0.93	C13	H13	0.93

The condensation of **2** with thiophene-2-carbaldehyde (**3b**) afforded a mixture of (*E*)- and (*Z*)-2-(benzothiazol-2'-ylthio)-3-(2''-thienyl) acrylonitriles in a ratio of 6:0.5 based on the ^1^H-NMR spectrum, which revealed, in addition to the expected aromatic signals, two downfield signals at δ_H_ 8.52 and 8.70 ppm assignable to the *E *and *Z*-vinylic protons. Upon recrystallization of the crude product from a mixture of ethanol and diethyl ether in a ratio of 3:2 one isomer **4b** was obtained. The ^1^H-NMR spectrum of the crystallized product showed the vinylic proton resonance at δ_H_ 8.52 ppm. In order to determine the configuration of the obtained isomer, different NMR experiments have been carried out such as COSY, HSQC and NOE experiments. The NOE experiments showed a strong NOE coupling between H-3 and (vinylic-H) and the thiophene protons H-3'' and H-4'' and no coupling between the benzothiazole and the thienyl ring protons, indicating that the benzothiazole and the thienyl rings are on opposite sides of the double bond, confirming that compound **4b **exists in the *E*-configuration.

**Table 6:** Bond angles (o) for compound **4a**.

atom	atom	atom	angle	atom	atom	atom	angle
C1	S1	C7	88.04(14)	C7	S2	C8	101.83(13)
C10	O1	C13	106.66(19)	C6	N1	C7	109.2(3)
S1	C1	C2	128.6(3)	S1	C1	C6	110.1(3)
C2	C1	C6	121.3(3)	C1	C2	C3	118.6(4)
C2	C3	C4	119.6(4)	C3	C4	C5	122.5(4)
C4	C5	C6	119.9(4)	N1	C6	C1	115.2(3)
N1	C6	C5	126.9(3)	C1	C6	C5	118.0(3)
S1	C7	S2	121.46(15)	S1	C7	N1	117.5(3)
S2	C7	N1	121.0(3)	S2	C8	C9	120.82(19)
S2	C8	C14	114.9(2)	C9	C8	C14	123.9(3)
C8	C9	C10	129.9(3)	O1	C10	C9	119.7(3)
O1	C10	C11	107.9(2)	C9	C10	C11	132.4(3)
C10	C11	C12	107.7(3)	C11	C12	C13	106.6(3)
O1	C13	C12	111.2(3)	N2	C14	C8	174.9(3)F

On the other hand, condensation of **2** with either benzaldehyde or *para*-substituted benzaldehydes **3c–e** in refluxing ethanol containing a catalytic amount of piperidine, afforded a mixture of *E* and *Z* isomers of 3-aryl-2*-*(benzothiazol-2'-ylthio) acrylonitriles **3c–e** in a ratio of 7:2 based on the ^1^H-NMR spectra of the crude products. The chemical shifts of the ^1^H- and ^13^C- spectra were assigned on the basis of the proton-proton and the carbon-proton coupling patterns observed in the COSY and HSQC spectra. The ^1^H-NMR spectra display vinylic proton singlets in the δ_H_ 8.12–8.29 ppm region. The chemical shifts for the carbon-carbon double bond are observed at δ_C_ 91–97 and at δ_C_ 157–158 ppm for the α- and β carbon, respectively. Moreover, Nuclear Overhauser Effect (NOE) experiments were run to establish the stereo-orientation for either the *E* or *Z* isomers of 2-(benzothiazol-2'-ylthio)-3-(4''-methoxyphenyl)acylonitrile **4d**. On irradiating the singlet of the vinylic proton at δ_H_ 8.17 ppm the doublet of H-2'' of phenyl ring at δ_H_ 8.00 ppm was enhanced. On irradiating the doublet signal of the phenyl ring H-3'' at δ_H_ 7.15 ppm both the doublet of the phenyl ring H-2'' and the *para*-methoxy group protons at δ_H_ 8.00 and 3.87 ppm, respectively, were enhanced, while the benzothiazole protons showed no effect, indicating that the benzothiazole and the 4-methoxyphenyl rings are on opposite sides of the double bond, as required by an *E*-form.

**Scheme 3:**
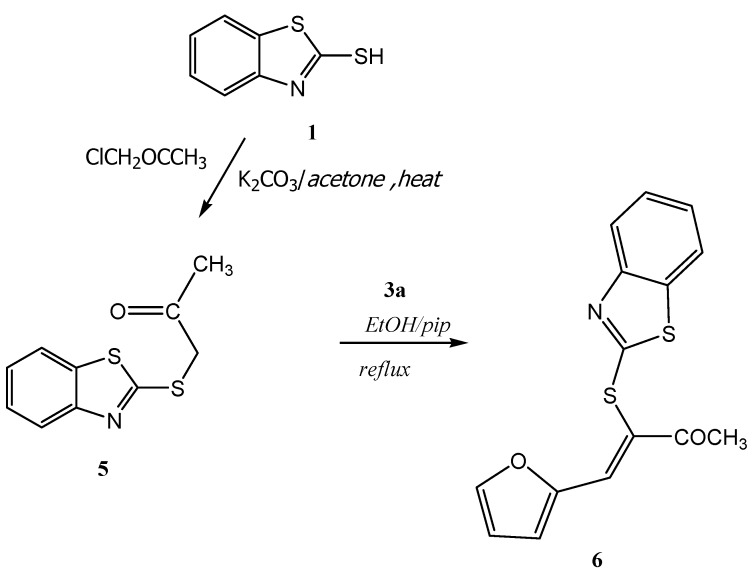
Synthesis of compound **6**.

**Figure 4:**
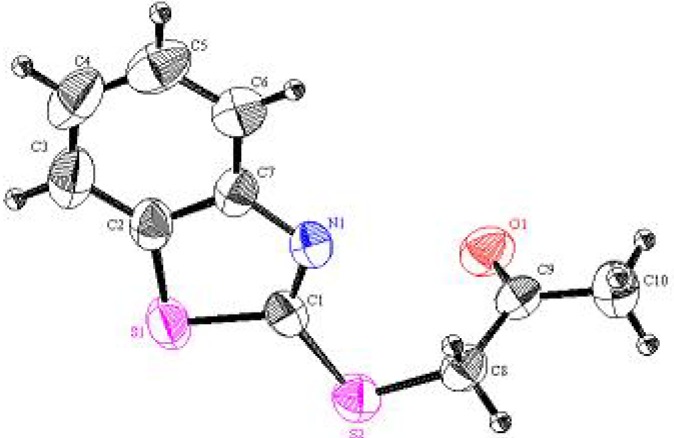
Perspective view and atom labeling of the X-ray structure of **5**.

In a similar manner, the 1-(benzothiazol-2'-ylthio)propan-2-one (**5**) used in our experiments has been recently prepared in an excellent yield by treatment of 2-mercaptobenzothiazole (**1**) with α-chloroacetone in refluxing acetone containing anhydrous potassium carbonate [15]. The structure of **5** was unambiguously confirmed by X-ray crystallography [19] (*cf*. Scheme 3, Figure 4 and Table 7, Table 8, Table 9). Condensation of **5** with the furan-2-carbaldehyde in refluxing ethanol containing a catalytic amount of piperidine afforded 3-(benzothiazol-2'-ylthio)-4-(furan-2''yl)-3-buten-2-one in excellent yield (*cf*. Scheme 3). The structure of the isolated product was confirmed on the basis of its elemental analysis and spectral data (see Experimental section).

**Table 7:** Bond lengths (Å) for compound **5**.

atom	atom	distance	atom	atom	distance
S1	C1	1.758(3)	S1	C2	1.730(4)
S2	C1	1.738(3)	S2	C8	1.785(3)
O1	C9	1.206(4)	N1	C1	1.289(4)
N1	C7	1.392(4)	C2	C3	1.387(5)
C2	C7	1.410(4)	C3	C4	1.369(6)
C4	C5	1.393(7)	C5	C6	1.371(5)
C6	C7	1.389(5)	C8	C9	1.501(4)
C9	C10	1.494(5)			

**Table 8:** Bond lengths involving hydrogen's (Å) for compound **5**.

atom	atom	distance	atom	atom	distance
C3	H3	0.930	C4	H4	0.930
C5	H5	0.930	C6	H6	0.930
C8	H8A	0.970	C8	H8B	0.970
C10	H10A	0.960	C10	H10B	0.960
C10	H10C	0.960			

**Table 9:** Bond angles (^o^) for compound **5**.

atom	atom	atom	angle	atom	atom	atom angle	
C1	S1	C2	88.71(14)	C1	S2	C8	101.10(14)
C1	N1	C7	110.6(3)	S1	C1	S2	116.94(16)
S1	C1	N1	116.3(3)	S2	C1	N1	126.8(2)
S1	C2	C3	129.9(3)	S1	C2	C7	109.6(3)
C3	C2	C7	120.5(3)	C2	C3	C4	118.8(4)
C3	C4	C5	120.8(4)	C4	C5	C6	121.3(4)
C5	C6	C7	118.8(4)	N1	C7	C2	114.8(3)
N1	C7	C6	125.4(3)	C2	C7	C6	119.8(3)
S2	C8	C9	116.2(3)	O1	C9	C8	122.8(3)
O1	C9	C10	122.3(3)	C8	C9	C10	114.9(3)

**Figure 5:**
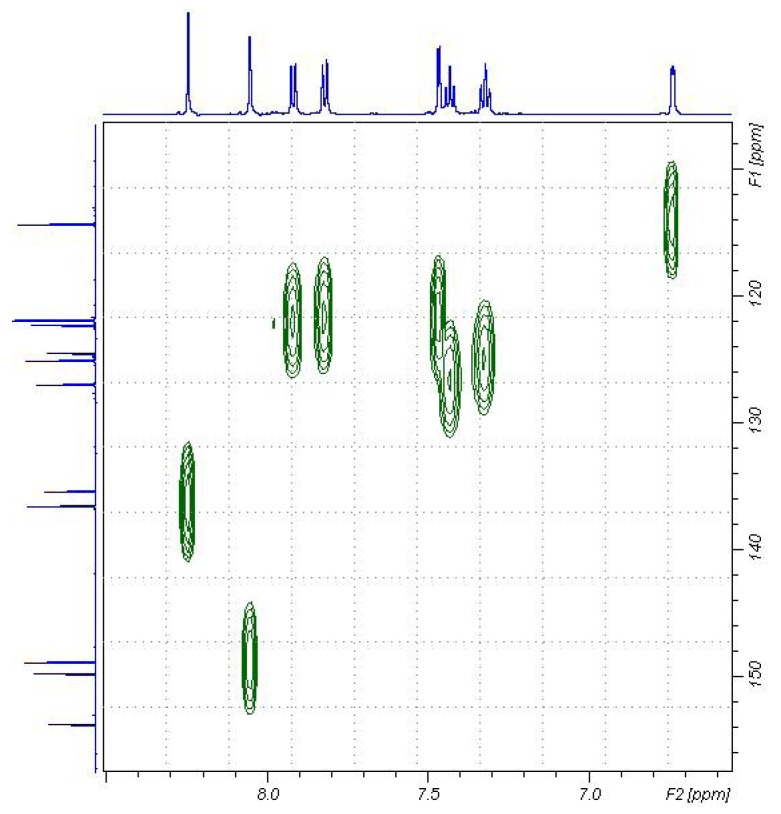
^13^C-HSQC spectra for the compound **6** in DMSO-d_6_.

**Figure 6:**
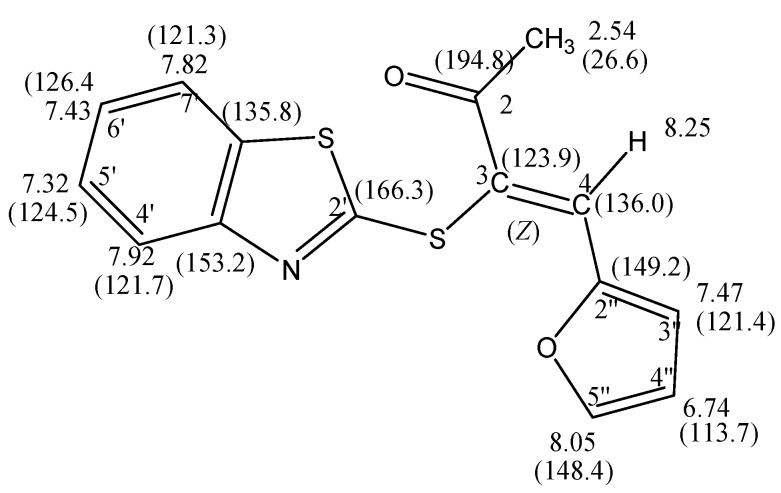
The complete assignment of H^1^ and ^13^C chemical shifts for **6** based on the COSY and HSQC experiments.

The mass spectrum of **6** revealed a molecular ion peak [M^+^]. with *m/z* 301 The effect of the conjugatation of the carbonyl group with the carbon-carbon double bond reduces the frequency of the absorption at υ_max_ 1662cm^−1^. It is considered lower than the isolated carbonyl group in compound **5**, which revealed a carbonyl stretching band at υ_max_ 1720 cm^−1^ [15]. The chemical shift of protons for **6** were assigned using COSY (correlation spectroscopy) measurements which provided the proton-proton coupling. The ^1^H-NMR spectrum showed a resonance at δ_H_ 8.25 ppm corresponding to H-4 of the vinylic proton. Moreover, the ^13^C-NMR chemical shift assignments were straightforward using HSQC (Heteronuclear Quantum Coherence) measurements (*cf*. Figure 5). The ^13^C-NMR spectrum of the reaction product revealed a low field signal at δ_C _194.8 that corresponds to the carbonyl carbon. Also were revealed two low field signals at ca. δ_C_ 148 and 136 ppm corresponding to a carbon coupled with a proton. As in the furan ring system, the carbon resonating at ca. δ_C_ 148 ppm corresponds to C-5'', while the carbon resonating at ca. δ_C _136 ppm corresponds to the vinylic carbon C-4. The complete assignment of H^1^ and ^13^C chemical shifts for **6** are presented in Figure 6.

Moreover, the configuration of the product **6** was assigned as the *Z*-isomer based on Nuclear Overhauser Effect (NOE) experiments; on irradiating the methyl proton at δ_H_ 2.54 ppm only the signal at δ_H_ 8.25 ppm for the vinylic proton was enhanced and there is no effect on the benzothiazole protons or furyl protons, which indicate that acetyl group and the vinylic proton are on the same side of the carbon 3,4 double bond as numbered, therefore it has the *Z* configuration. On irradiating the vinylic H-4 at δ_H_ 8.25 ppm both the singlet and doublet signals at δ_H_ 2.54 and 7.47 ppm, respectively, were enhanced. The signal at δ_H_ 2.54 ppm corresponds to the acetyl group, while the doublet signal at δ_H_ 7.47 ppm corresponds to the furyl system H-3". On other hand, on irradiating the doublet signal of the H-4' proton at δ_H_ 7.92 ppm only the triplet signal at δ_H_ 7.32 ppm was enhanced. These two signals correspond to the benzothiazole H-4' and H-5' protons. On irradiating the triplet signal H-4'' at δ_H_ 6.74 ppm both the H-5'' and H-3'' signals at δ_H_ 8.05 and 7.47 ppm, respectively, were enhanced. The NOE experiments show that the *Z*-isomer of compound **6** was preferred over the *E*-isomer indicating that the furyl and 2-benzothiazol-2-thio nuclei are on the same sides of the double bond, but away from each other in space.

**Scheme 4:**
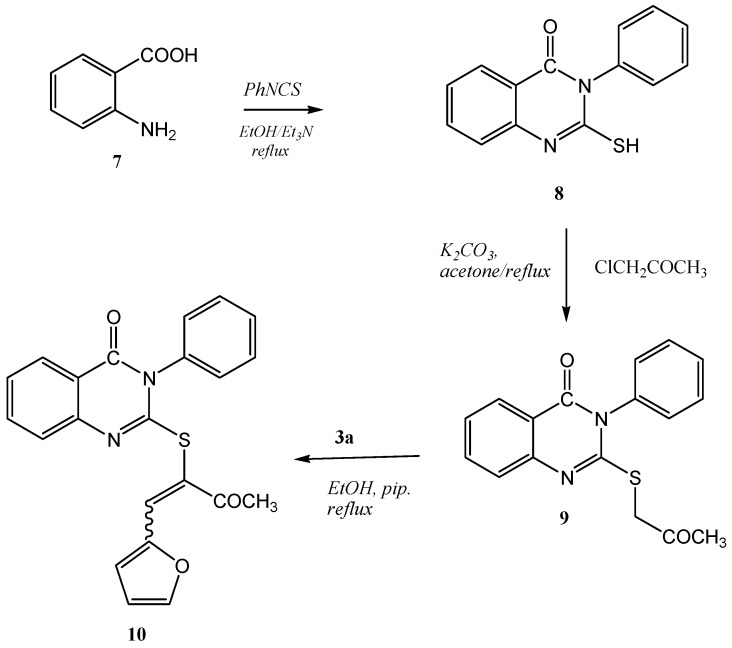
Synthesis of **9** and **10**.

Thus it can be concluded that Knoevenagel condensation of 2-(benzothiazol-2-ylthio) acetonitrile with furan-2-carbaldehyde leads preferentially to *(E)-2-(benzothiazol-2'-ylthio)-3-(furan-2''-yl) acrylonitrile * (**4a**). In this conformation the lone pair electrons of the sulfur atom can conjugated with the π orbital of the double bond, and thereby stabilize the molecule. That is why this conformation is the most stable conformer. On the other hand, the Knoevenagel condensation of 3-(benzothiazl-2'-ylthio) propan-2-one with furan-2-carbaldehyde lead preferentially to *(Z)-3-(benzothiazol-2'-ylthio)-4-(furan-2''-yl)-3-buten-2-one*. By changing the size and the geometry of the group attached to the α-carbon from a cyano group to an acetyl group, the conformation changes from *E* to *Z*. One may therefore conclude that under these circumstances the most *E*-isomer is unlikely to be the most stable conformer, because of the steric strain between the acetyl group and furan ring. The steric strain makes this conformation energetically highly unfavorable in the *E*-isomer so the conformer changes from *E* to *Z*. On the other hand, 2-(2'-oxopropylthio)-3-phenylquinazolin-4(3*H*)-one (**9**) was readily prepared in good yield by the treatment of 2-mercapto-3-phenylquinazolin-4(3*H*)-one (**8**) [20] with α-chloroacetone in refluxing acetone containing potassium carbonate. The structure was established on the basis of its spectral data. The mass spectrum of the reaction product showed a molecular ion peak at *m/z* 310. The ^1^H-NMR spectrum revealed, in addition to aromatic signals, two upfield singlets at δ_H_ 2.33 and 4.06 ppm, assignable to methyl and methylene protons, respectively. Moreover, the ^13^C-NMR spectrum showed two downfield signals at δ_C_ 202.5 and 161.1 ppm. The signal at δ_C_ 202.5 ppm corresponds to the ketone carbonyl carbon, while that at δ_C_ 161.1 ppm corresponds to the cyclic amide carbon .The data are therefore consistent with structure **9** (*cf*. Scheme 4).

**Figure 7:**
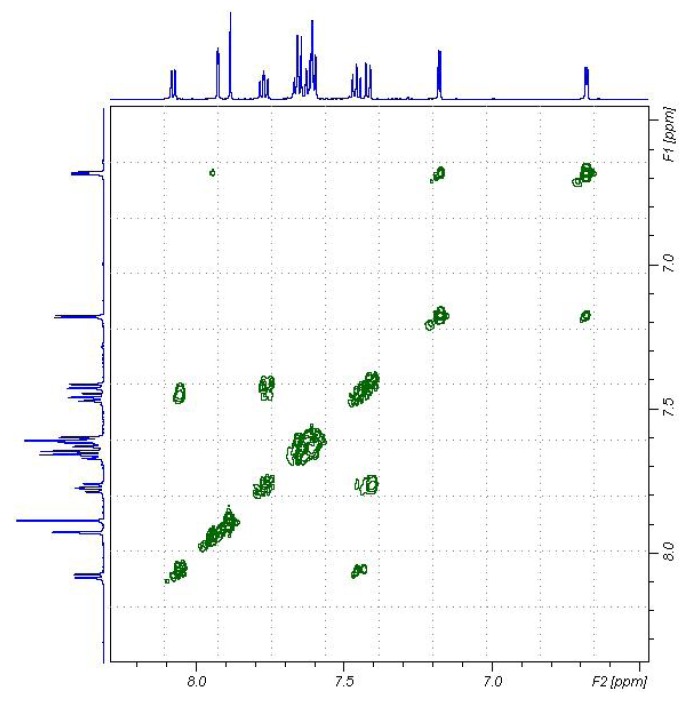
^13^C-HSQC spectra for the compound **10** in DMSO-d_6_.

**Figure 8:**
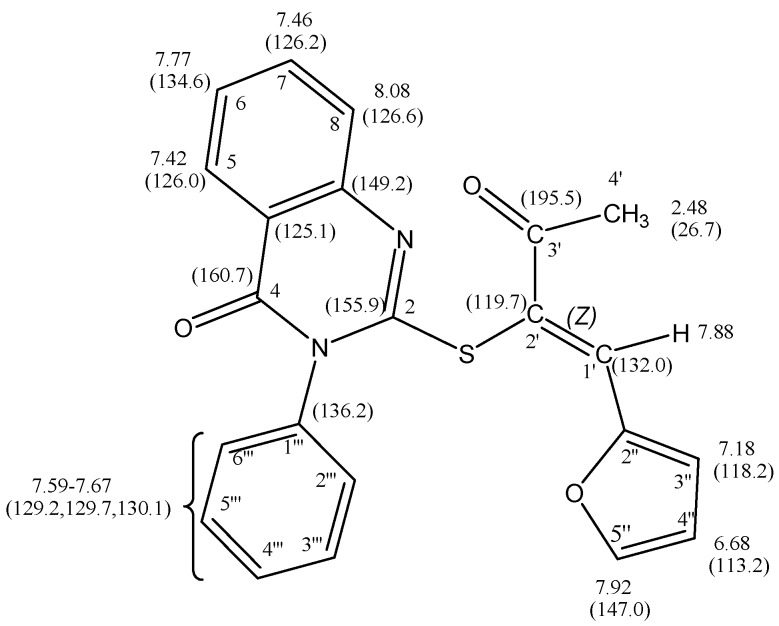
The complete assignment of H^1^ and ^13^C chemical shifts for **10** based on the COSY and HSQC experiments.

Condensation of 2-(2'-oxopropylthio)-3-phenylquinazolin-4(3*H*)-one (**9**) with furan-2-carbaldehyde (**3a**) afforded a product which could have been either *E* or *Z*-isomer of α,β-unsaturated ketone **10** (*cf*. Scheme 4). The structure of α,β-unsaturated ketone **10** was deduced from its elementanal anaylsis and spectra data. The mass spectrum revealed a molecular ion peak (M^+^) with *m/z* 388. The chemical shifts of the protons for **10** were assigned using COSY (correlation spectroscopy) measurements which provided the proton-proton coupling. The ^1^H-NMR revealed in addition to an aromatic multiplet, a singlet for a vinylic proton (H-1') which appears at low field at ca. δ_H_ 7.88 ppm. Moreover, the ^13^C-NMR chemical shift assignments were straightforward using HSQC (Heteronuclear Single Quantum Coherence) measurements (*cf*. Figure 7). The ^13^C-NMR spectrum for **10** is characterized by two signals at δ_C_ 132.0 and δ_C_ 119.7 ppm for the α,β-unsaturated ketone group. The vinylic carbons at the higher frequency is the one coupled with a proton, while the other one is a disubstituted sp^2^ carbon. The complete assignment of H^1^ and ^13^C chemical shift for **10** are presented in Figure 8.

Moreover, the configuration of the product **10** was assigned as the *Z*-isomer based on Nuclear Overhauser Effect (NOE) experiments; on irradiating the methyl proton at δ_H_ 2.48 ppm the vinylic proton signal at δ_H_ 7.88 ppm was enhanced. There is no effect on the 3-phenylquinazolin-4(3*H*)-one or furan protons, which indicates that the acetyl group and the vinylic proton are on the same side of the C1',2' double bond as numbered, which therefore has *Z* configuration. On irradiating the vinylic H'-1 at δ_H_ 7.88 ppm the methyl signal at δ_H_ 2.48 ppm was enhanced.

**Figure 9:**
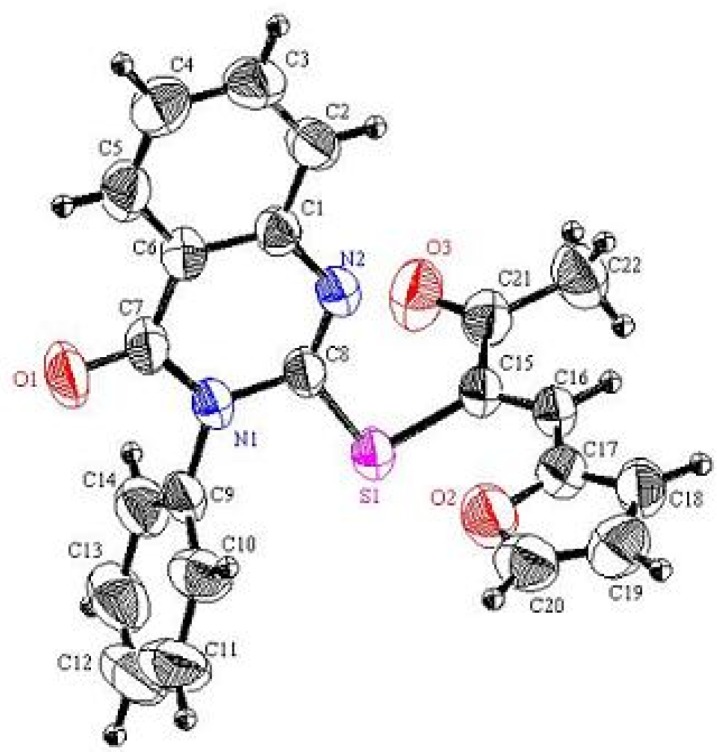
Perspective view and atom labeling of the X-ray structure of **10**.

On the other hand irradiation of the H-8 proton at δ_H_ 8.08 ppm has no effect, confirming thatthe 3-phenylquinazolin-4(3*H*)-one moiety and 2'-furyl groups are on same sides of the double bondas required by a *Z-*form. The structure of **10** was also confirmed by X-ray crystallography [21] (*cf*. Figure 9 and Table 10, Table 11, Table 12).

**Table 10:** Bond lengths (Å) for compound **10**.

atom	atom	distance	atom	atom	distance
S1	C8	1.7609(19)	S1	C15	1.7687(19)
O1	C7	1.219(3)	O2	C17	1.350(3)
O2	C20	1.398(4)	O3	C21	1.216(3)
N1	C7	1.402(3)	N1	C8	1.400(3)
N1	C9	1.440(3)	N2	C1	1.389(3)
N2	C8	1.285(3)	C1	C2	1.404(3)
C1	C6	1.397(3)	C2	C3	1.382(4)
C3	C4	1.388(4)	C4	C5	1.365(4)
C5	C6	1.399(4)	C6	C7	1.456(3)
C9	C10	1.378(4)	C9	C14	1.382(4)
C10	C11	1.393(5)	C11	C12	1.374(6)
C12	C13	1.354(6)	C13	C14	1.384(5)
C15	C16	1.354(3)	C15	C21	1.483(3)
C16	C17	1.424(3)	C17	C18	1.345(4)
C18	C19	1.385(4)	C19	C20	1.324(5)
C21	C22	1.499(4)			

**Table 11:** Bond lengths involving hydrogen's (Å) for compound **10**.

atom	atom	distance atom atom distance			
C2	H2	0.930	C3	H3	0.930
C4	H4	0.930	C5	H5	0.930
C10	H10	0.930	C11	H11	0.930
C12	H12	0.930	C13	H13	0.930
C14	H14	0.930	C16	H16	0.930
C18	H18	0.930	C19	H19	0.930
C20	H20	0.930	C22	H22A	0.960
C22	H22B	0.960	C22	H22C	0.960

**Table 12:** Bond angles (o) for compound **10**.

atom	atom	atom	angle	atom	atom	atom	angle
C8	S1	C15	100.89(9)	C17	O2	C20	106.1(2)
C7	N1	C8	121.46(16)	C7	N1	C9	118.33(15)
C8	N1	C9	120.21(15)	C1	N2	C8	117.26(15)
N2	C1	C2	118.64(16)	N2	C1	C6	122.50(16)
C2	C1	C6	118.86(17)	C1	C2	C3	119.7(2)
C2	C3	C4	120.9(3)	C3	C4	C5	119.9(3)
C4	C5	C6	120.3(3)	C1	C6	C5	120.22(19)
C1	C6	C7	119.83(17)	C5	C6	C7	119.91(18)
O1	C7	N1	120.0(2)	O1	C7	C6	125.9(2)
N1	C7	C6	114.04(16)	S1	C8	N1	113.15(13)
S1	C8	N2	122.04(14)	N1	C8	N2	124.81(17)
N1	C9	C10	119.6(2)	N1	C9	C14	118.9(2)
C10	C9	C14	121.5(3)	C9	C10	C11	118.6(3)
C10	C11	C12	119.7(3)	C11	C12	C13	121.2(4)
C12	C13	C14	120.5(3)	C9	C14	C13	118.6(3)
S1	C15	C16	121.83(16)	S1	C15	C21	116.04(14)
C16	C15	C21	122.06(18)	C15	C16	C17	131.01(19)
O2	C17	C16	125.4(2)	O2	C17	C18	109.4(2)
C16	C17	C18	125.1(2)	C17	C18	C19	107.8(3)
C18	C19	C20	107.6(3)	O2	C20	C19	109.1(3)
O3	C21	C15	120.5(2)	O3	C21	C22	119.7(3)
C15	C21	C22	119.8(2)				

In particular, we were interested in the reasons for the predominance of the s-*cis* conformation of the *Z* configuration and the *E*/*Z *determination of the prepared novel derivatives of 2-(benzothiazol-2'-ylthio)-3-arylacrylonitrile, 3-(benzothiazol-2'-ylthio)-4-(furan-2''-yl)-3-buten-2-one and 2-(1-(furan-2''-yl)-3-oxobut-1-en-2-ylthio)3-phenylquinazolin-4(3*H*)-one in accordance with expectations, it was ascertained that s-*trans* conformations were more stable than s-*cis* conformations for both the *E* and *Z* molecular configurations. Thus the disparity displayed here where s-*cis* conformations in case of compound **6** dominated over the s-*trans* conformation was of interest and one supposition was that electronic interactions could be responsible, e.g., by delocalization of a sulfur lone electron pair with the unsaturated segments residing in the newly formed heterocyclic ring and attendant side-chains or through hyperconjugation. The s-*cis* conformational preference over s-*trans* in a structurally similar system has been reported previously [22] and an electronic cause was also postulated, though that system differed significantly in the distribution of unsaturation. It is obvious to note that compounds **4c–e** are predominantly in the *E/Z* form and this is due to the large range of mesomeric effects due to large conjugations in the phenyl group present in such compounds. However, the existence of compounds **4a** and **4b** in the *E* form is due to the existence of the heterocyclic, furan and thiophene rings, respectively, where less conjugation occurs in such compounds.

## 3. Experimental

### General

Melting points are reported uncorrected and were determined on a Gallenkamp apparatus. The Infrared spectra were recorded on a Jasco FT/IR-6300 FT-IR using KBr disks. ^1^H-NMR and ^13^C-NMR spectra were measured on a Bruker DPX 400 MHz and Bruker AVANCE ΙΙ 600 MHz spectrometers, with DMSO-d_6_ or CDCl_3_ as solvent using TMS as an internal standard. The methods used for the purpose of NMR assignment were COSY, HSQC and HMBC. The chemical shifts are expressed as δ unit in parts per million (ppm) and TMS = 0.00 ppm. The following abbreviation are used: s = singlet, d = doublet, t = triplet; q = quartet; m = multiple; br. = broad. Mass spectra were measured on GC/MS DFS, THERMO instrument. Microanalyses were performed on a CHNS-Vario Micro Cube analyzer, Single crystal X-ray crystallography was perfomed using a Rigaku Rapid ΙΙ located at the Chemistry Department of Kuwait University. Compound **5** was prepared according to our recent reference [15] and its X-ray data was reported in reference [19].

*2-(Benzothiazol-2-ylthio) acetonitrile * (**2**): A mixture of **1** (1.67 g, 10.0 mmol), chloroacetonitrile (0.63 g, 10.0 mmol), and anhydrous potassium carbonate (1.38 g, 10.0 mmol) in acetone (100 mL), were heated in water bath for 2 h. The solvent was then evaporated under reduced pressure. The solid product, so formed, was collected by filtration and crystallized from ethanol as brown crystals. Yield: 1.85 g (90%), mp. 70–72 °C; FT-IR: ν_max/cm_^−1^: 2243 (CN); ^1^H-NMR (DMSO-d_6_): δ_H_ 4.58 (s, 2H, CH_2_), 7.43 (t, 1H, *J* = 8.0 Hz, H-5'), 7.52 (t, 1H, *J* = 8.0 Hz , H-6'), 7.95 (d, 1H, *J* = 8.0 Hz, H-7'), 8.08 (d, 1H, *J* = 8.0 Hz, H-4'); ^13^C-NMR (DMSO-d_6_): δ_C_ 163.8 (C-2'), 152.7 (C-3a'), 135.6 (C-7a'), 127.1 (C-6'), 125.5 (C-5'), 122.6 (C-4'), 122.0 (C-7'), 117.9 (CN), 18.6 (CH_2_) ppm; MS *m/z* (%) 206 [M^+^, 100%]. *Anal*. Calcd. for C_9_H_6_N_2_S_2_ (206.28): C, 52.40; H, 2.93; N, 13.57%. Found: C, 52.29; H, 3.19; N, 13.45%.

*General Procedure for Synthesis of 3-Aryl-2-(benzothiazol-2'-ylthio) acrylonitriles* **4a–e**. A mixture of **2** (2.06 g, 10.0 mmol) and aromatic aldehydes **3a–e** (10.0 mmol) in ethanol (20 mL) containing a few drops of piperidine was refluxed for 4 h. The reaction was allowed to cool to room temperature for 24 h. The solid product so formed was collected by filtration and crystallized from the appropriate solvent.

*(E)-2-(Benzothiazol-2'-ylthio)-3-(furan-2''-yl) acrylonitrile* (**4a**): This compound was crystallized from ethanol as brown crystals. Yield: 2.24g (79%) yield, mp. 134–136 °C; FT-ir: ν_max/cm_^−1^: 2209 (CN); ^1^H-NMR (DMSO-d_6_): 6.84 (dd, 1H, *J* = 3.6 & 1.2 Hz, H-4''), 7.30 (d, 1H, *J* = 3.6 Hz, H-3''), 7.43 (t, *J* = 8.2 Hz, H-5'), 7.51 (t, 1H, *J* = 8.0 Hz, H-6'), 7.94 (d, 1H, *J* = 8.2 Hz, H-7'), 8.06 (s, 1H, H-3), 8.07 (d, 1H, *J* = 8.0 Hz, H-4'), 8.15 (d, 1H, *J* = 1.2 Hz, H-5''); ^13^C-NMR (DMSO-d_6_): δ_C_ 164.6 (C-2'), 153.1 (3a'), 148.8 (C-5"), 148.2 (C-2''), 142.2 (C-3), 135.3 (C-7a'), 126.8 (C-6'), 125.2 (C-5'), 122.1 (C-4'), 121.9 (C-7'), 121.6 (C-3''), 116.9 (CN), 113.8 (C-4''), 91.9 (C-2) ppm. MS *m/z* (%) 284 [M^+^, 18%]. *Anal*. Calcd. for C_14_H_8_N_2_OS_2_ (284.36): C, 59.13; H, 2.84; N, 9.85; S, 22.55%. Found: C, 58.95; H, 2.81; N, 10.07; S, 22.56%.

*(E)-2-(Benzothiazol-2'-ylthio)-3-(thiophen-2''-yl) acrylonitrile *(**4b**). This compound was crystallized from a 3:2 mixture of ethanol/diethyl ether as yellow crystals. Yield: 2.4 g (88%), mp. 98–100 °C; FT-IR: ν_max/cm_^−1^: 2210 (CN); ^1^H-NMR (DMSO-d_6_): δ_H_ 7.35 (dd, 1H, *J* = 4.8 & 3.5 Hz, H-4''), 7.43 (t, 1H, *J* = 7.6 Hz, H-5'), 7.52 (t, 1H, *J* = 7.6 Hz, H-6'), 7.89 (d, 1H, *J* = 3.5 Hz, H-3''), 7.95 (d, 1H, *J* = 8.0 Hz, H-7'), 8.09 (d, 1H, *J* = 8.0 Hz, H-4'), 8.14 (d, 1H, *J* = 4.8 Hz, H-5''), 8.52 ppm (s, 1H, H-3); ^13^C-NMR (DMSO-d_6_): δ_C_ 164.8 (C-2'), 153.2 (C-3a'), 150.4 (C-3), 137.5 (C-3''), 135.9 (C-2''), 135.3 (C-7a'), 135.1 (C-5''), 128.6 (C-4''), 126.8 (C-6'), 125.2 (C-5'), 122.1 (C-4'), 121.9 (C-7'), 117.1 (CN), 92.3 (C-3) ppm; MS *m/z *(%) 300 [M^+^, 14%]. *Anal*. Calcd. for C_14_H_8_N_2_S_3_ (300.42): C, 55.97; H, 2.68; N, 9.32; S, 32.01%. Found: C, 56.04; H, 2.52; N, 9.54; S, 32.08%.

*(E)-2-(Benzothiazol-2'-ylthio)-3-phenyl acrylonitrile* (**4c**). This compound was crystallized from a 2:1 mixture of ethanol/diethyl ether as yellow crystals. Yield: 2.08 g (71%) , mp. 103–105 °C; FT-IR: ν_max/cm_^−1^: 2206 (CN); ^1^H-NMR (DMSO-d_6_): δ_H_ 7.38 (t, 1H, *J* = 7.8 Hz, H-5'), 7.49 (t, 1H, *J* = 8.4 Hz, H-6' ), 7.58–7.63 (m, 3H, H-3'', H-4'' & H-5''), 7.96 (d, 1H, *J* = 8.4 Hz, H-7), 7.97 (d, 2H, *J* = 8.4 Hz, H-2''& H-6''), 8.10 (d, 1H, *J* = 8.0 Hz, H-4'), 8.29 ppm (s, 1H vinylic-H); ^13^C-NMR (DMSO-d_6_): δ_C _163.7 (C-2'), 157.0 (C-3), 153.1 (C-3a'),135.4 (C-7a'), 132.6 (C-4''), 130.8 (C-1''), 129.5 (C-2'' & C-6''), 128.9 (C-3'' & C-5''),126.8 (C-6'),125.5 (C-5'), 122.2 (C-4'), 122.0 (C-7'), 116.8 (CN), 97.5 (C-2) ppm. MS *m/z *(%) 294 [M^+^, 83%]. *Anal*. Calcd. for C_16_H_10_N_2_S_2_, (294.39) requires: C, 65.28 ; H, 3.42; N, 9.52; S, 21.78%. Found: C, 64.98; H, 3.25; N, 9.73; S, 22.03%.

*(E)-2-(Benzothiazol-2'-ylthio)-3-(4''-methoxyphenyl) acrylonitrile* (**4d**). This compound was crystallized from ethanol as brown crystals. Yield: 2.62 g (81%), mp. 95–97 °C. FT-IR: ν_max/cm_^−1^: 2202 (CN); ^1^H-NMR (DMSO-d_6_): δ_H_ 3.87 (s, 3H, OCH_3_), 7.15 (d, 2H, *J* = 9.0 Hz, H-3'' & H-5''), 7.42 (t, 1H, *J* = 7.8 Hz, H-5'), 7.51 (t, 1H, *J* = 7.8 Hz, H-6'), 7.94 (d, 1H, *J* = 7.8 Hz, H-7'), 8.00 (d, 2H, *J* = 8.4 Hz, H-2’’ & H-6’’), 8.06 (d, 1H, *J* = 7.8 Hz, H-4'), 8.17 ppm (s, 1H, H-3); ^13^C-NMR (DMSO-d_6_): δ_C_ 165.0 (C-2'), 162.9 (C-4''), 157.3 (C-3), 153.3 (C-3a'), 135.3 (C-7a'), 132.1 (C-2'', C-6"), 126.7 (C-6'), 125.1 (C-5'), 124.9 (C-1''), 122.1 (C-4'), 121.9 (C-7'), 117.5 (CN), 114.8 (C-3'' & C-5"), 92.8 (C-2), 55.7 (OCH_3_) ppm; MS *m/z *(%) 324 [M^+^, 20% ]. *Anal*. Calcd. for C_17_H_12_N_2_OS_2_ (324.42): C, 62.94; H, 3.73; N, 8.63; S, 19.76%. Found: C, 63.06; H, 3.63; N, 8.87; S, 20.06%.

*(E)-2-(Benzothiazol-2'-ylthio)-3-(4''-hydroxyphenyl) acrylonitrile* (**4e**). This compound was crystallized from a 2:1 mixture of ethanol/diethyl ether as yellow crystals. Yield: 2.32 (75%), mp. 113–114 °C ; FT-IR: ν_max/cm_^−1^: 3417 (OH), 2200 (CN); ^1^H-NMR (DMSO-d_6_): δ_H_ 6.97 (d, 2H, *J* = 8.4 Hz, H-3'' & H-5''), 7.39 (t, 1H, *J* = 8.4 Hz, H-5'), 7.50 (t, 1H, *J* = 8.4 Hz, H-6'), 7.91 (d, 2H, *J* = 8.4 Hz, H-2'' & H-6''), 7.93 (d, 1H, *J* = 8.4, Hz, H-7'), 8.06 (d, 1H, *J* = 8.4 Hz, H-4'), 8.12 (s, 1H, H-3), 9.05 (bs., 1H, OH, D_2_O exchangeable); ^13^C-NMR (DMSO-d_6_): δ_C _165.6 (C-2'), 162.2 (C-4''), 158.0 (C-3), 153.3 (C-3a'), 135.2 (C-7a'), 132.5 (C-2'' & C-6''), 126.7 (C-6'), 125.0 (C-5'), 123.0 (C-1''), 122.1 (C-4'), 121.8 (C-7'), 117.8 (CN), 116.3 (C-3'' & C-5''), 91.2 (C-2) ppm; MS *m/z *(%) 310 [M^+^, 44%]. *Anal*. Calcd. for C_16_H_10_N_2_OS_2_ (310.39): C, 61.91; H, 3.25 ; N, 9.03; S, 20.66%. Found: C, 61.63; H, 3.40; N, 9.27; S, 20.92%.

*(Z)-3-(Benzothiazol-2'-ylthio)-4-(furan-2''-yl)-3-buten-2-one *(**6**): A mixture of **5** (2.33 g, 10.0 mmol) and furan-2-carbaldehyde **3a** (10.0 mmol) in ethanol (20 mL) containing a few drops of piperidine was refluxed to 4 h. The reaction was allowed to cool to room temperature for 24 h. The solid product so formed was collected by filtration and crystallized from the ethanol as yellow crystals. Yield: 2.16 g (72%) yield, mp. 85–87 °C ; FT-IR: ν_max/cm_^−1^: 1662 (CO); ^1^H-NMR (DMSO-d_6_): 2.54 (s, 3H, CH_3_), 6.74 (dd, 1H, *J* = 3.6 & 1.2 Hz, H-4''), 7.32 (t, 1H, *J* = 8.0 Hz, H-5'), 7.43 (t, 1H, *J* = 8.0 Hz, H-6'), 7.47 (d, 1H, *J* = 3.6 Hz, H-3''), 7.82 (d, 1H, *J* = 8.0 Hz, H-7'), 7.92 (d, 1H, *J* = 8.0 Hz, H-4'), 8.05 (d, 1H, *J* = 1.6 Hz, H-5''), 8.25 (s, 1H, H-4); ^13^C-NMR (DMSO-d_6_): δ_C_ 194.8 (C-2), 166.3 (C-2'), 153.2 (C-3a'), 149.2 (C-2''), 148.4 (C-5''), 136.0 (C-4), 135.8 (C-7a'),126.4 (C-6'), 124.5 (C-5'), 123.9 (C-3), 121.7 (C-4'), 121.4 (C-3''), 121.3 (C-7') ,113.7 (C-4''), 26.6 (CH_3_) ppm; MS *m/z *(%) 301 [M^+^, 30%]. *Anal*. Calcd. for C_15_H_11_NO_2_S_2_ (301.38): C, 59.78; H, 3.67; N, 4.64%. Found: C, 59.41; H, 3.68; N, 4.80%.

*3-(2'-Oxopropylthio)-3-phenylquinazolin-4(3H)-one* (**9**): A mixture **8** (2.54 g, 10.0 mmol), chloro-acetone (0.79 g, 10.0 mmol), and anhydrous potassium carbonate (1.38 g, 10.0 mmol) in acetone (100 mL) were heated in water bath for 2 h. The solvent was then evaporated under reduced pressure. The solid product, so formed, was collected by filtration and crystallized from ethanol as yellow crystals. Yield: 2.4 g (79%), mp. 140–142 °C. FT-IR: ν_max/cm_^−1^: 1725 (CO ketone), 1683 (CO amide); ^1^H-NMR (DMSO-d_6_): δ_H_ 2.33 (s, 3H, CH_3_) ,4.06 (s, 2H, CH_2_), 7.29 (d, 1 Hz, *J* = 7.6 Hz, H-5), 7.35 (t, 1H, *J* = 7.6 Hz, H-7), 7.63–7.41 (m, 5H, phenyl–H), 7.81 (t, 1H, *J* = 8.0 Hz, H-6), 8.08 (d, 1H, *J* = 7.6Hz, H-8); ^13^C-NMR (DMSO-d_6_): δ_C_ 202.5 (C-2'), 161.1 (C-4), 157.3 (C-2), 147.5 (C-8a), 136.4 (C-6), 135.4 (C-1''), 130.4, 129.8, 129.5 (phenyl carbons), 127.1 (C-8), 126.5 (C-7), 126.4 (C-5), 123.9 (C-4a), 42.6 (C-1'), 28.7 (CH_3_) ppm; MS *m/z* (%) 310 [M^+^, 12%]. *Anal*. Calcd. for C_17_H_14_N_2_O_2_S(310.37): C, 65.79, H, 4.55, N, 9.03%. Found: C, 65.65, H, 4.30, N, 9.06%.

*(Z)-2-(1'-(Furan-2''-yl)-3'-oxobut-1''-en-2-ylthio)-3-phenylquinazolin-4(3H)-one* (**10**): A mixture of **9** (3.10 g, 10 mmol) and **3a** (0.83 g, 10 mmol) in ethanol (20 mL) containing a few drops of piperidine was refluxed for 4 h. The reaction was allowed to cool to room temperature for 24 h. The solid product so formed was collected by filtration and crystallized from ethanol as brown crystals. Yield: 2.83 g (73%) yield, mp. 190–192 °C; FT-IR: ν_max/cm_^−1^: 1675 (CO ketone), 1610 (CO amide); ^1^H-NMR (DMSO-d_6_): 2.48 (s, 3H, CH_3_), 6.68 (dd, 1H, *J* = 3.6 Hz & 1.8 Hz, H-4''), 7.18 (d, 1H, *J* = 3.6 Hz, H-3''), 7.42 (d, 1H, *J* = 8.0 Hz, H-5), 7.46 (t, 1H, *J* = 8.0 Hz, H-7), 7.67–7.59 (m, 5H, phenyl–H),7.77 (t, 1H, *J* = 8.0 Hz, H-6), 7.88 (s, 1H, H-1'), 7.92 (d, 1H, *J* = 1.8 Hz, H-5''), 8.08 (d, 1H, *J* = 8.0 Hz, H-8) ppm; ^13^C-NMR (DMSO-d_6_): δ_C_ 195.5 (C-3'), 160.7 (C-4), 155.9 (C-2), 149.2 (C-8a), 147.0 (C-5''), 146.8 (C-2''), 136.2 (C-1'''), 134.9 (C-6), 132.0 (C-1', vinylic–H), 130.1, 129.7, 129.2 (phenyl carbons), 126.6 (C-8), 126.2 (C-7), 126.0 (C-5), 125.1 (C-4a), 119.7 (C-2'), 118.2 (C-3''), 113.2 (C-4''), 26.7 (CH_3_) ppm; MS *m/z* (%) 388 [M^+^, 82]. *Anal*. Calcd. for C_22_H_16_N_2_O_3_S( 388.44): C, 68.02; H, 4.15; N, 7.21; S, 8.25%. Found: C, 67.78; H, 4.26; N, 7.16, S, 8.32%.

## 4. Conclusions

Knoevenagel condensation of 2-(benzothiazol-2'-ylthio) acrylonitrile (**2**) with aromatic benzaldehydes **3a–e** leads preferentially to *E*-isomers. The 3-aryl-2-(benzothiazol-2'-ylthio) acrylonitriles **4a–e** were characterized by spectroscopic measurements. Condensation of furan-2-carbaldehyde with either 1-(benzothiazol-2'-ylthio)propan-2-one or with 2-(2'-oxopropylthio)-3-phenylquinazolin-4(3H)-one afforded compounds **6** and **10** respectively. The condensation products **6** and **10** were characterized by spectroscopic measurements and were shown to be the *Z*-isomers.
